# Non-alcoholic fatty liver disease in type 1 diabetes: Prevalence and pathophysiology

**DOI:** 10.3389/fendo.2022.1031633

**Published:** 2022-12-01

**Authors:** Plator Memaj, François R. Jornayvaz

**Affiliations:** ^1^ Division of Endocrinology, Diabetes, Nutrition and Therapeutic Patient Education, Department of Medicine, Geneva University Hospitals, Geneva, Switzerland; ^2^ Diabetes Center, Faculty of Medicine, Geneva University, Geneva, Switzerland; ^3^ Department of Cell Physiology and Metabolism, Faculty of Medicine, Geneva University, Geneva, Switzerland

**Keywords:** NAFLD, type 1 diabetes, glycogenic hepatopathy, prevalence, pathophysiology

## Abstract

Non-alcoholic fatty liver disease (NAFLD) is the most frequent chronic liver disease in the general population with a global prevalence of 25%. It is often associated with metabolic syndrome and type 2 diabetes, as insulin resistance and hyperinsulinemia are known to be favoring factors. Recent studies have described growing incidence of NAFLD in type 1 diabetes (T1D) as well. Although increasing prevalence of metabolic syndrome in these patients seems to explain part of this increase in NAFLD, other underlying mechanisms may participate in the emergence of NAFLD. Notably, some genetic factors are more associated with fatty liver disease, but their prevalence in T1D has not been evaluated. Moreover, oxidative stress, poor glucose control and long-lasting hyperglycemia, as well as exogenous insulin administration play an important role in intrahepatic fat homeostasis. The main differential diagnosis of NAFLD in T1D is glycogenic hepatopathy, which needs to be considered mostly in T1D patients with poor glycemic control. This article aims to review the prevalence and pathophysiology of NAFLD in T1D and open perspectives for clinicians taking care of T1D patients with potential hepatopathy.

## Introduction

Non-alcoholic fatty liver disease (NAFLD) is characterized by the accumulation of lipids in the liver, particularly in the absence of high-risk alcohol consumption. It has seen its prevalence increase steadily for several years due to the global epidemic of overweight and obesity (1, 2). Insulin resistance is a pathological process very frequently associated with NAFLD and explains a very strong association of this condition with diabetes (2).

In recent years, NAFLD in patients with type 1 diabetes (T1D) rises a particular interest due to its apparent higher prevalence (3–5). The rising prevalence of metabolic syndrome in T1D due to unhealthy lifestyle is one important explanation of this increase of NAFLD in these individuals (6), but other underlying biologic mechanisms found in T1D tend to favor liver fat accumulation.

By understanding these mechanisms, we can not only have a better comprehension of NAFLD development, but this can also help us find ways to slow, stop and prevent fatty liver disease in patients with T1D.

## Methodology

A literature review was realized using PubMed, Google Scholar and Web of Science including several studies which were linked to the association between NAFLD or MAFLD and T1D. Medical Subject Headings terms such as “Non-alcoholic fatty liver disease”, “Metabolic-dysfunction associated fatty liver disease”, “Glycogenic Hepatopathy”, “Liver disease”, “NASH”, “Steatohepatitis” were associated with “Type 1 Diabetes”. The different articles were analyzed and selected according to their abstract relevance. Similar articles suggested by the research sites were also taken into consideration and selected. In total, this review was based on the study of 62 different articles. The articles were all restricted to English language.

### NAFLD and diabetes: Definition and generality

NAFLD encompasses several pathologies affecting the liver ranging from simple hepatic steatosis to non-alcoholic steatohepatitis (NASH), and subsequently cirrhosis which is the most severe form of NAFLD. Cirrhosis may lead to hepatocellular carcinoma. The differences between these stages of liver damage can be seen on analysis of a histological section after performing a liver biopsy, which remains an invasive procedure associated with potential morbi-mortality (1). Over the past four decades, NAFLD has become the most prevalent chronic liver disease affecting approximately 25% of the adult population worldwide (1). NAFLD prevalence is even higher in type 2 diabetic (T2D) patients, reaching about 55%, and up to 90% in obese patients with a body mass index (BMI) above 40 kg/m^2^ (7). Given its increasing prevalence, NAFLD is the most rapidly increasing cause of liver-related mortality (7). There is no specific approved treatment for this disease and its pathophysiological complexity represents a challenge for the development of potential therapeutic targets. Lifestyle changes remain the best way to prevent and treat the disease. NAFLD is usually associated with metabolic syndrome including T2D and obesity (8, 9). Additionally, this disease is also associated with other illnesses and factors such as dyslipidemia, hypertension, genetic and environmental factors, notably lack of exercise and unhealthy food intake (10). Regarding the mortality of patients with NAFLD in the general population, various studies have shown contradictory results with, on one hand, a slight increase in mortality (all causes combined) in patients with NAFLD compared to the general population and, on the other hand, other studies showed no association between mortality and NAFLD (11, 12). Although NAFLD increases the risk of developing cirrhosis or hepatocellular carcinoma, the main cause of death in these patients remains cardiovascular diseases followed by extrahepatic malignancies (13–15).

T1D is an autoimmune disease characterized by the destruction of beta cells resulting in the cessation of insulin production (16). T1D is mainly diagnosed in childhood or adolescence (under the age of 18) but can also be diagnosed in adults. The risk of developing T1D is extremely low in the general population (0.4%) but increases in the presence of risk factors such as a T1D in a first-degree relative (parent or brother/sister) or the presence of self-specific antibodies (17, 18). Typically, the autoantibodies sought in T1D are anti-GAD (glutamic acid decarboxylase), IA2 (islet antigen 2), ZnT8 (Zinc transporter protein member 8) and Islets of Langerhans (18). The initial clinical presentation, the family history of T1D and the age at diagnosis help in the diagnosis even if T1D can be diagnosed at a later age (Latent Autoimmune Diabetes in Adults, LADA). The initial clinical presentation is classically in the form of diabetic ketoacidosis with an acidic pH (<7.35), increased blood glucose and presence of plasmatic ketone bodies. However, it should be considered that the number of patients affected by T2D is increasing in the young population (19) making the diagnosis of T1D less obvious in young patients. Usually, the disease presents in 3 stages: The first stage consists in the destruction of beta cells with normal blood glucose levels and no symptoms. The second stage is characterized by the presence of hyperglycemia, but the patient usually remains asymptomatic. Finally, the third stage is the time of diagnosis characterized by the presence of symptoms such as polydipsia, polyuria, weight loss, dehydration, etc. (16). It is common to find, in addition to T1D, other autoimmune diseases such as Hashimoto’s thyroiditis, celiac disease, pernicious anemia, etc. Therefore, screening for other autoimmune diseases is recommended in patients with T1D (16). Apart from the fact that type 1 diabetics are usually thinner and younger than type 2 diabetics and that they have positive antibodies, unlike type 2 diabetics, another way to distinguish them is the measurement of C-peptide in the blood. The latter determines the insulin reserve that the pancreas produces and is very low in T1D (20). The only treatment for T1D remains the subcutaneous injection of long-acting and short-acting insulins. With the technological advances of recent years, patients with T1D can benefit from insulin pump systems that will, when coupled to a blood glucose sensor and a correction algorithm, adjust the dose of insulin given continuously to keep blood glucose within the targets. Recent studies are looking at possible treatments that could prevent or slow the progression of T1D in people at risk. Teplizumab is a monoclonal antibody that appears to slow the progression of T1D in newly diagnosed patients. Its action seems to protect the remaining beta cells against autoantibodies (16). This area is still unexplored and there is a lot of research to do in immunotherapy for T1D.

### Diagnosis of NAFLD

Simple hepatic steatosis in NAFLD can be assessed by histology following a biopsy or by imaging like ultrasound imaging or magnetic resonance imaging (MRI). Liver biopsy is the gold standard for diagnosing and characterizing liver histologic alterations in NAFLD (3, 21). Histologically, NAFLD is defined as the presence of at least 5% hepatic steatosis without evidence of hepatocellular injury such as hepatocyte ballooning, whereas NASH is characterized by the presence of hepatocellular injury with lobular inflammation and hepatocellular ballooning (22, 23).

Liver biopsy is an invasive procedure, and imaging is therefore more frequently used to diagnose NAFLD. Hepatic fat content can be evaluated using conventional imaging such as ultrasonography, computed tomography (CT) and MRI. Nonetheless, these conventional imaging are limited for different reasons, such as lack of sensitivity and specificity (for ultrasonography and CT), lack of objectivity (for ultrasonography and MRI), radiation safety issues (CT) and different confounding factors (for all conventional imaging) (24). One of the main confounding factors is differential diagnosis, particularly hepatic glycogenesis and glycogenic hepatopathy (25). Nevertheless, recent advances in imaging such as multi-parametric MRI can help detect hepatic fat more efficiently. The multi-parametric MRI with, notably, the proton density fat fraction allows to overcome these limitations and has become a virtual liver biopsy method which can help avoid unnecessary biopsies and can also be used for the follow-up during therapy (24). Given the high and growing prevalence in NAFLD, this new imaging method can turn out to be crucial.

### NAFLD prevalence in type 1 diabetes

As discussed above, there is a clear and known link between NAFLD and T2D. However, in recent years, there has been a significant increase in type 1 diabetic patients affected by NAFLD, although studies on this subject are scarce (25). It is known that the prevalence of NAFLD in type 2 diabetics (55.5%) is more than two times higher than in the general population (25%) (26). The association between T2D and NAFLD has been known and studied for several decades and the interest in the subject is significant, while the association with T1D has only been recently explored.

A recent systematic review and meta-analysis included twenty studies about the prevalence of NAFLD in T1D. In total, 3’901 subjects were included in this study. Overall, 19.3% of subjects with T1D had NAFLD, whereas NAFLD prevalence was 22% in type 1 diabetic adults only, which is less than in the general population (3). However, there were significant differences between the 20 studies included in this meta-analysis, depending on the way NAFLD was diagnosed. Three ways were used to diagnose NAFLD: Ultrasound, MRI and liver biopsy. When looked separately, NAFLD was found in 27.1% of subjects using ultrasound, in 8.6% using MRI and 19.3% using liver biopsy, the latter being the gold standard (3). To interpret these results in the context of the general population, other parameters must be taken in consideration. Indeed, patients with T1D are younger and mostly non obese.

Another study compared NAFLD prevalence in type 1 diabetics, type 2 diabetics and healthy individuals who were matched for age and BMI. This study showed that only 4.7% (6 out of 128) of type 1 diabetics had NAFLD, versus 13.4% (9 out 67) of healthy individuals, versus 62.8% (166 out of 264) of type 2 diabetics (4). In this study, the diagnostic modality used to evaluate liver fat content was MRI and hepatic steatosis was defined as liver fat content > 5.5%. In a more recent meta-analysis, the prevalence of NAFLD in lean/nonobese healthy individuals was reported to range from 10.2% to 15.7% (5).

When compared to the above discussed meta-analysis, which showed that 19.3% of subjects (including children, adolescents and adults) with T1D had NAFLD, this is in contrast with the second one which showed that 4.7% of people with T1D had NAFLD. Considering the sample sizes, 3901 individuals in the first study versus 128 in the second study, we can presume that the statistical power of the first study is much higher and therefore potentially more representative of NAFLD prevalence in T1D.

Another study looked into etiologic factors of NAFLD development in patients with T1D and T2D using transient elastography to diagnose NAFLD and to assess the presence or absence of advanced liver fibrosis. This study reported that NAFLD prevalence in T1D patients (N=150) was 20% (N=30) and 76% (N=76) in T2D patients (N=100) (27). Advanced liver fibrosis was found in 2% (N=3) of T1D patients and in 22% (N= 22) of T2D patients. Hepatic steatosis was estimated by controlled attenuation parameter and hepatic fibrosis by liver stiffness measurement using transient elastography (27). Interestingly, larger waist circumference, higher BMI and presence of metabolic syndrome were all positively associated with the presence of NAFLD in both groups, whereas insulin sensitivity, calculated with estimated glucose disposal rate and SEARCH estimated insulin sensitivity, were negatively associated with the presence of NAFLD (27).

In conclusion, we can say that most studies report a higher prevalence of NAFLD in T1D, but further analyses must be done to support this statement. Therefore, it remains difficult to establish whether individuals with T1D are more likely to develop NAFLD and a lot of limitations can explain the difficulty to prove a clear link between those two diseases, one of them being the diagnostic modality used.

### NAFLD pathophysiology in T1D and T2D

NAFLD pathophysiology in T2D might differ in some points from NAFLD pathophysiology in T1D but it can help understand how T1D may contribute to the development of NAFLD (**Table 1**). In T2D, insulin resistance plays a key role in the development of NAFLD (2). Additionally, some lipid intermediates found in the development of NAFLD, such as diacylglycerols and ceramides, are more likely to cause hepatic insulin resistance than others, thus alimenting a vicious cycle leading to the increase of NAFLD (28). Insulin resistance is in fact associated with increased circulating free fatty acids and ectopic lipid accumulation in the liver, which can further promote inflammation and endoplasmic reticulum stress, participating also in this vicious cycle of the insulin resistance state (29). Inflammation seems to play an important role in both insulin resistance and NAFLD, with inflammatory mediators such as cytokines and adipokines playing a primordial role not only in inflammation but also in metabolic energy balance and immune response (30). Oxidative stress, which is caused by the excessive presence of intracellular reactive oxygen species (ROS), also plays a key role in the development of NAFLD. NADPH Oxidase (NOX) enzymes are the main producers of ROS, and it has been shown that their increased activity is linked to NAFLD and insulin resistance due to hepatic lipid overload (31, 32). Also, it is known that obesity and unhealthy food habits lead to excessive production of ROS by creating an imbalance between ROS production and elimination, and therefore participate even more in the development of insulin resistance and liver tissue damage participating in the vicious cycle (31). Subjects with NAFLD have in general lower plasma adiponectin concentrations than individuals without NAFLD and it is known that adiponectin plays an anti-inflammatory role and improves hepatic insulin sensitivity (33).

**Table 1:** Comparison of NAFLD Pathophysiology mechanisms between in T1D and T2D.

NAFLD pathophysiological mechanisms	T1D	T2D
Insulin resistance	+	+++
Altered dynamic of insulin delivery	++	–
Altered insulin clearance	+++	+
Relative insulin resistance in hepatocytes	++	+++
SREBP and ChREBP activation by hyperglycemic state and high fructose intake	+	++
Hyperglucagonemia and hepatic glucagon resistance (worsened by amylin deficiency)	+++	–
Low GLP-1 blood concentration	+	++

(-: unlikely; +: not unlikely; ++: likely; +++: very likely). T1D, type 1 diabetes; T2D, type 2 diabetes; SREBP, sterol regulatory element-binding proteins; ChREBP, carbohydrate response element-binding protein; GLP-1, glucagon-like peptide-1.

In T1D, it has been shown that insulin resistance and obesity are increasing with time and these described mechanisms in T2D may be likely to occur in T1D (6). Since T1D only relies on exogenous insulin subcutaneous administration, one of the factors influencing the pathophysiology of NAFLD development in these individuals is the altered dynamic of insulin delivery and of insulin clearance. Hyperinsulinemia in patients with NAFLD appears to be much more correlated with impaired insulin clearance than with increased insulin secretion (34). A recent study assessed the role of metabolic determinants of NAFLD in T1D individuals. Poor glycemic control (HbA1c > 7%) doubled the risk of NAFLD, and the prevalence in patients with BMI > 25 kg/m^2^ was higher (66%) than the overall NAFLD prevalence (47%). Interestingly, 37% of the lean individuals (BMI < 25 kg/m^2^) had NAFLD and this was correlated with total insulin dose. This study shows in patients with T1D the potential importance of exogenous injected insulin and the crucial impact of obesity in the development of NAFLD (35).

CEACAM1 (Carcinoembryonic antigen-related cell adhesion molecule 1) is a cell transmembrane protein playing a key role in insulin degradation and thus its clearance and is abundantly found in hepatocytes to help regulation of insulin homeostasis. CEACAM1 mediates excess insulin removal through its phosphorylation induced by the ligand activated insulin receptor to maintain normal insulinemia (36). There are two main mechanisms that can compromise CEACAM1 phosphorylation and action: hyperinsulinemia and impaired pulsatility of insulin secretion. As a reminder, it has been known for a long time now that beta cells release insulin in two phases: following blood glucose increase with a peak secretion, then followed by a slower release to maximal secretion levels until glycemia is back to normal (37). Considering the importance of insulin secretion pulsatility for CEACAM1’s efficiency to clear insulin, continuous high insulinemia exposure not only downregulates insulin receptor density, but also downregulates insulin clearance, therefore increasing insulinemia and insulin resistance. The less variation in insulin concentration, the more insulin is needed to be effective.

Poor glucose control leads to hyperglycemia which then increases expression of GLUT-2, a glucose transporter in hepatocytes. In this state of insulin resistance and hyperinsulinemia with hyperglycemia, hepatic lipogenesis is upregulated because of the increase of lipogenic substrate (glucose) availability through GLUT-2 increase and because of the lipogenic effect of insulin (*de novo* lipogenesis) (25). Because of the high blood glucose level and insulin action, glycogen synthesis is enhanced but when glycogen synthesis pathways are saturated due to long-lasting hyperglycemia exposure, glucose is shunted to lipogenic pathways thus favoring NAFLD development (23, 38).

In T1D, subcutaneous insulin injections are required to maintain normal blood glucose levels and it is unlikely that all injected insulin reaches the liver through the portal vein as in endogenous insulin production, then implying a relative state of insulin resistance and increased insulin requirement (25, 39) (23, 40).

Intrahepatic lipogenesis is enhanced by insulin notably by increasing sterol regulatory element-binding proteins (SREBPs) in hepatocytes and stimulating them (41). These proteins not only help for cholesterol, free fatty acids, triglycerides and phospholipids synthesis and uptake, but are also essential for enzymes expression that are required for lipogenesis (42). SREBP-1c protein, which is upregulated by hyperglycemia, is crucial for glucokinase, liver-type pyruvate kinase (LPK), fatty acid synthase (FAS), and acetyl-CoA-carboxylase (ACC) expression, which all participate in the increase of lipogenesis (43). LPK gene transcription is also stimulated by another transcription factor called ChREBP (carbohydrate response element-binding protein) but is only highly activated in hyperglycemic state without the influence of insulinemia (44). We can then hypothesize that SREBP and ChREBP are important factors and contributors for the development of NAFLD in T1D.

These factors are also activated by chronic fructose consumption usually found in individuals with metabolic syndrome and T2D (45). However, fructose consumption by T1D individuals is very common given the potentially frequent hypoglycemias experienced by these individuals. To correct their low blood sugar level, they use sugar-rich beverages which are often fructose-rich nutrients such as sodas/soft drinks, fruit juices or processed food. This behavior can occur every day for a lot of T1D and contribute not only to weight gain or obesity, but also to the activation of lipogenesis leading therefore to NAFLD susceptibility (40, 46).

T1D is also associated with other pancreatic hormones abnormalities such as hyperglucagonemia. Glucagon is a hormone secreted by alpha cells to counteract the effects of insulin to stabilize blood glucose level. It is usually suppressed by hyperglycemia and by paracrine insulin production but not by exogenous insulin administration, explaining in part hyperglucagonemia seen in T1D (47). Another cause of hyperglucagonemia in T1D is the lack of amylin secretion usually produced by beta cells simultaneously with insulin in response to nutrient stimuli. Amylin suppresses glucagon production in response to postprandial glucose increase, avoiding hepatic glucose production, and slows gastric emptying, avoiding glucose excursions (48). In normal individuals, glucagon increases hepatic lipolysis with free fatty acids oxidation, and suppresses lipogenesis, thus having likely a protective effect against fat accumulation in the liver (49). Nonetheless, hepatic glucagon resistance has been found in patients with NAFLD, thereby promoting fat accumulation in the liver and hyperglycemia through lack of neoglucogenesis inhibition (50). Therefore, hyperglucagonemia found in T1D could contribute to the development and worsening of NAFLD, although there is not enough evidence yet.

Another hormone that rose a lot of interest these recent years is glucagon-like peptide-1 (GLP-1). GLP-1 is an incretin hormone secreted by intestinal L cells upon food intake with effects on satiety, glycemia and gastric emptying. It has been shown that GLP-1 agonists reduce liver fat accumulation and reduce NASH activity (51). GLP-1 agonists have also shown to upregulate CEACAM1 transcription, thus increasing insulin clearance, which helps protecting the liver from insulin resistance and from fat deposition (36). In some studies, GLP-1 blood concentrations have been shown to be lower in patients with T1D and as such could also be one of the factors contributing to NAFLD development (40, 52–54). This hypothesis should be further studied since other work seems to support the fact that there is no significant difference in GLP-1 blood concentrations between T1D patients and the general population (55, 56).

### Glycogenic hepatopathy: A differential diagnosis

One of the main differential diagnoses of NAFLD that can be seen on imaging, especially ultrasonography, is glycogenic hepatopathy. This is a rare condition characterized by the accumulation of glycogen in the hepatocytes, mostly affecting children and adolescents with poorly controlled T1D (25). Initially, glycogenic hepatopathy was considered to be part of Mauriac syndrome, which is a complication of badly controlled T1D with delayed puberty, dwarfism, cushingoid features and liver enlargement due to glycogen deposition (57). However, glycogenic hepatopathy was later dissociated from Mauriac syndrome and characterized by glycogen accumulation in hepatocytes due to poor glycemic control without any other features of Mauriac syndrome (58). To diagnose glycogenic hepatopathy, a liver biopsy is required (59). Imaging is also used to help diagnose glycogenic hepatopathy, for example using ultrasonography. The main difficulty with ultrasonography remains its poor specificity due to similarities found in both NAFLD and glycogenic hepatopathy even though both diseases can coexist at the same time considering that their cause is identical: poor glucose control (25). Since MRI can distinguish fat from glycogen, it can be used to distinguish these two pathologies much more efficiently than ultrasonography or CT. To distinguish one from the other, there are some biological and clinical characteristics that can help, such as abdominal discomfort and elevation of liver enzymes, both found more often in glycogenic hepatopathy (**Table 2**) (60).

**Table 2:** Comparison between Glycogenic Hepatopathy and Non-Alcoholic Fatty Liver Disease (NAFLD).

	Glycogenic Hepatopathy	Non-Alcoholic Fatty Liver Disease
Age at onset	Mostly children and adolescents	Mostly Adults
Uncontrolled T1D with extremely poor glucose control	Yes	Not necessarily
Symptoms	Present (abdominal discomfort)	Uncommon
Signs	Tender hepatomegaly	Ascites in advanced NAFLD
Liver Enzymes	Mild to severe elevation	No or mild elevation (mostly alanine transaminase)
Ultrasonography findings	Hyperechogenic: due to glycogen deposition	Hyperechogenic: due to fat deposition
Magnetic Resonance Imaging findings	Absence of steatosis (no difference in intensities)	Presence of steatosis (difference in intensities)
Diagnosis: Gold Standard	Histology (liver biopsy)	Histology (liver biopsy)

T1D, type 1 diabetes.

## Discussion

NAFLD in T1D has become a subject of interest these recent years with more studies assessing a potential link between these diseases, since NAFLD is a rising disease that we still know little about despite more studies now being published in this field. Nonetheless, it seems very likely that there is a causative link between T1D and NAFLD, and exploring this association with further studies will help understand and treat NAFLD in T1D. It is not totally clear if patients with T1D are more susceptible to develop NAFLD as studies seem to be contradictory about whether NAFLD prevalence in T1D is higher than in the general population or not (3–5). Although most studies seem to show a higher prevalence of NAFLD in T1D, further work must be done to support this statement.

The main limitation of these studies assessing NAFLD prevalence in T1D remains the diagnostic modality used. Since the gold standard to diagnose NAFLD remains liver biopsy, but is expensive and risky to perform in a large population and since there is no blood biomarkers specific enough for NAFLD, imaging diagnosis remains the best way to diagnose NAFLD for now with multi parametric MRI being considered as a virtual biopsy with great specificity and sensitivity (24). However, given the cost of MRI, applying it to a large number of individuals will be a limitation for further studies.

NAFLD encompasses a whole spectrum of liver injuries including NASH. However, there is currently very little data on NASH prevalence in patients with T1D. It has been shown in a study that NASH has been histologically diagnosed in 20.4% of T1D individuals (10 out of 49 individuals) whereas it has been diagnosed in 44.4% of the T2D individuals (20 out of 45 individuals) (61). The T1D cohort in this study was younger but diabetes duration before liver biopsy was longer in the T2D cohort. A recent study in 2021 compared 30 T1D patients with 37 T2D patients in order to assess the relationship between hepatic energy metabolism and diabetes-related NAFLD. This study showed that, as expected, T2D individuals had higher hepatocellular lipid content (38% in T2D *vs.* 7% in T1D) and higher insulin resistance despite similar glycemic control. The follow-up after 5 years showed that hepatocellular lipid content doubled in T2D individuals with an increase of visceral adipose tissue, increasing the prevalence of NAFLD up to 70%. This was correlated with insulin resistance, and hepatic energy metabolism, estimated with γATP and inorganic phosphate (Pi) concentrations, was impaired in both individuals but significantly more in T2D individuals (17% *vs.* 10% in T1D). Altogether, this study suggests that fat tissue mass and liver mitochondria have an important role in the development of NAFLD in patients with diabetes (62). This can suggest the important role of excessive visceral adipose tissue in NAFLD and NASH emergence. Since there is only little data regarding NASH prevalence in T1D, further work is therefore required to specifically address this question.

Another area yet to be explored is searching for biological blood biomarkers that would be highly specific for NAFLD in T1D. A potential candidate is CEACAM1, which is known to be downregulated in NAFLD and upregulated with GLP-1 analogs (36). CEACAM1, a transmembrane protein acting in hepatocytes to get rid of insulin excess hence limiting insulin resistance, has been shown to be lower in T1D and could be the link between NAFLD and T1D (36). Another interesting biomarker that can help diagnose NAFLD is an elevated alanine transaminase (ALT) blood concentration. Indeed, elevated ALT concentration is frequently encountered in T1D-associated NAFLD (63). Nevertheless, elevated liver enzymes can have many causes and raised liver enzymes are not necessarily present in NAFLD (25).

Since CEACAM1 plays a crucial role in insulin resistance and in NAFLD development, we can hypothesize that pharmacologically upregulating CEACAM1 could be a promising therapeutic approach for the treatment of NAFLD in T1D. As described above, GLP-1 analogs along with PPARγ (peroxisome proliferator-activated receptor γ) agonists have both shown good potential since they both increase CEACAM1 transcription. Other potential therapeutic targets include molecules such as GIP (gastric inhibitory polypeptide) analogs, which are also part of the incretin hormones family like GLP-1 analogs, or a combination of both GLP-1 analog and GIP analog such as the dual agonist tirzepatide. Nevertheless, studies in this area are still needed to evaluate the potential of this group of molecules on NAFLD, not only in T2D, but also in T1D. Another potential therapy could be amylin analogs since amylin in T1D is lacking and it was demonstrated that pramlintide, a synthetic amylin analog, showed improvement in metabolic control (25, 64). A retrospective analysis showed that short-chain fatty acids can influence gut barrier health and have positive effects not only on NAFLD, but also on T1D. Short-chain fatty acids, especially butyrate, seem to prevent the destruction of gut barrier by maintaining it and strengthening it. They also participate in the regulation of gut microbiota and immune cells, and for all these reasons short-chain fatty acids represent another promising potential therapy for NAFLD and T1D (65).

## Conclusion

There are several important points to keep in mind when it comes to NAFLD and T1D: the diagnostic modality used for NAFLD diagnosis is very important since NAFLD is difficult to diagnose without histological analysis and conventional imaging is often insufficient (24). Glycogenic hepatopathy is radiologically similar to NAFLD mostly in ultrasonography and it is important to remember the other differences that help distinguish them, such as elevated liver enzymes and abdominal discomfort, usually not found in NAFLD (28). New imaging techniques such as multi parametric MRI show promising results but remain costly and therefore represents a major limitation (24). Even though NAFLD in T1D can be partly explained by the increase in obesity and metabolic syndrome in T1D subjects, some other pathways different from the ones found in metabolic syndrome and T2D may be the key to understand the relation between T1D and NAFLD development (25, 44, 46). Relative hepatic insulin resistance caused by impaired insulin pulsatility and impaired insulin clearance, as well as hyperglucagonemia, both play a crucial role in NAFLD development and are both present in T1D (36, 47). GLP-1 agonists, amylin agonists and short-chain fatty acids have shown promising results in the treatment of NAFLD but must be further investigated, notably in T1D (51, 64, 65).

## Author contributions

Both authors contributed equally to the article and approved the submitted version.

## Funding

Open access funding was provided by the University of Geneva.

## Conflict of interest

The authors declare that the research was conducted in the absence of any commercial or financial relationships that could be construed as a potential conflict of interest.

## Publisher’s note

All claims expressed in this article are solely those of the authors and do not necessarily represent those of their affiliated organizations, or those of the publisher, the editors and the reviewers. Any product that may be evaluated in this article, or claim that may be made by its manufacturer, is not guaranteed or endorsed by the publisher.
